# Neuroprotection in Diabetes Retinal Disease: An Unmet Medical Need

**DOI:** 10.3390/ijms27020901

**Published:** 2026-01-16

**Authors:** Hugo Ramos, Olga Simó-Servat

**Affiliations:** 1 Diabetes and Metabolism Research Unit, Vall d’Hebron Research Institute, Universitat Autònoma de Barcelona, 08035 Barcelona, Spain; 2 Centro de Investigación Biomédica en Red de Diabetes y Enfermedades Metabólicas Asociadas (CIBERDEM), Instituto de Salud Carlos III (ICSIII), 28029 Madrid, Spain

**Keywords:** diabetes, diabetic retinopathy, retina, neurovascular unit, neurodegeneration, neuroprotection

## Abstract

Diabetic retinopathy (DR) has been classically considered a microvascular disease with all diagnostic and therapeutic resources focusing on its vascular components. However, during the past years, the obtained evidence highlighted the critical pathogenic role of early neuronal impairment redefining DR as a neurovascular complication. Retinal neurodegeneration is triggered by chronic hyperglycemia, which activates harmful biochemical pathways that lead to oxidative stress, metabolic overload, glutamate excitotoxicity, inflammation, and neurotrophic factor deficiency. These drivers of neurodegeneration can precede detectable vascular abnormalities. Simultaneously, endothelial injury, pericyte loss, and breakdown of the blood–retinal barrier compromise neurovascular unit integrity and establish a damaging cyclic loop in which neuronal and vascular dysfunctions reinforce each other. The interindividual variability of these processes highlights the need to properly redefine patient phenotyping by using advanced imaging and functional biomarkers. This would allow early detection of neurodegeneration and patient subtype classification. Nonetheless, translation of therapies based on neuroprotection has been limited by classical focus on vascular impairment. To meet this need, several strategies are emerging, with the most promising being those delivered through innovative ocular routes such as topical formulations, sustained-release implants, or nanocarriers. Future advances will depend on proper guidance of these therapies by integrating personalized medicine with multimodal biomarkers.

## 1. Introduction

Diabetic retinopathy (DR) is one of the most frequent complications of diabetes and the leading cause of visual impairment and blindness among the working-age population in the most developed countries [1,2]. Classically, DR has been considered a microvascular disease, which was mainly characterized by vascular leakage, ischemia, and neovascularization [3]. In accordance with this, most current diagnostic and therapeutic approaches have been designed to address vascular abnormalities, including treatments such as laser photocoagulation or intravitreal injections against vascular endothelial growth factor (VEGF) [4].

Nevertheless, growing evidence indicates that retinal neurodegeneration is an early and critical event in the pathogenesis of diabetic retinal disease [5,6]. Deficits in the functionality of retinal neurons, particularly in ganglion and amacrine cells, occur before vascular lesions become detectable [5,6]. The main pathological mechanisms behind neurodegeneration are oxidative stress, excitotoxicity, chronic low-grade inflammation, and neurotrophic factor deficiency, all of which contribute to neuronal dysfunction, apoptosis, and visual function impairment [7,8,9]. For all these reasons, DR was redefined as a neurovascular complication [10].

Despite this emerging understanding, the neurodegenerative component of diabetic retinal disease remains under-recognized in clinical practice. There is a pressing need for more refined retinal phenotyping in patients with diabetes, incorporating advanced imaging modalities and functional biomarkers beyond microvascular imaging that can detect neurodegeneration at its earliest stages [11,12,13,14]. Such approaches will allow a more precise classification of disease subtypes, help identify patients at highest risk of progression, and open the door to personalized therapeutic strategies [12,13].

Neuroprotection represents a promising medical solution for the treatment of diabetic retinal disease; however, it remains an unmet medical need. While current therapies are predominantly focused on vascular impairment, novel therapeutic strategies that target neuronal survival pathways have the potential to transform the current treatment paradigm, especially those that can be administered via innovative non-invasive routes, such as topical eye drops [15].

This review aims to summarize the pathogenic mechanisms behind the neurodegenerative process that diabetes induces in the retina to outline and discuss the emerging neuroprotective strategies and to highlight the relevance of an improved retinal phenotyping in patients with diabetes. The review focuses on the earliest stages of diabetic retinal disease, in which neuronal dysfunction and neurovascular unit (NVU) impairment precede clinically detectable ischemia, neovascularization, and proliferative disease.

## 2. Pathogenic Mechanisms of Diabetes-Induced Retinal Neurodegeneration

Diabetic retinal disease is increasingly recognized as a condition where neuronal, glial, and vascular dysfunctions interact from the earliest stages [13,16]. Neurodegenerative changes may occur before or in parallel with classical microvascular anomalies and involve retinal ganglion cells (RGCs), amacrine cells, bipolar cells, photoreceptors, and their glia (Müller cells) [7,16]. The mechanisms that trigger the neurodegenerative process are multifactorial and interact with each other. Briefly, chronic hyperglycemia and metabolic stress promote oxidative stress, accumulation of advanced glycation end-products (AGEs), mitochondrial dysfunction, glutamate excitotoxicity, microglial activation, and loss of neurotrophic support, all of which contribute to progressive neuronal dysfunction and cell death [9,13,17,18,19].

Meanwhile, hyperglycemia damages endothelial cells and pericytes, causing the breakdown of the inner and outer blood–retinal barrier (BRB), capillary occlusion, and ischemia [7,13]. Hypoxia stimulates VEGF production, which leads to vascular leakage, macular edema, and neovascularization [20,21]. These vascular abnormalities, in turn, exacerbate the neurodegenerative process by worsening ischemia and inflammation, creating a vicious cycle. For that reason, DR is defined as a neurovascular disorder where vascular and neuronal dysfunctions evolve simultaneously and amplify each other, ultimately leading to progressive vision loss [7,22].

### 2.1. Hyperglycemia: Underlying Mechanism in Early Neuronal Impairment

Retinal neurons are considered one of the most metabolically demanding cell types in the body, as they require a continuous glucose supply to satisfy their high energy demands. Glycolysis is their most immediate and accessible source of adenosine triphosphate (ATP), independently of oxygen availability or intracellular compartmentalization [23,24,25]. This strong dependence on glucose metabolism is essential for visual function, but it makes retinal neurons highly vulnerable to glucose fluctuations. As a result, neuronal impairment is one of the earliest consequences of hyperglycemia in DR [26,27].

Under diabetic conditions, chronic hyperglycemia increases the glycolytic flux generating excess intermediates that trigger four major damaging pathways: protein kinase C (PKC), hexosamine, polyol, and advanced glycation end-products (AGEs) [28].

- PKC pathway: When glyceraldehyde-3-phosphate levels increase, diacylglycerol (DAG) synthesis is enhanced activating PKC isoforms (α, β, δ, ε). In neurons, PKC dysregulation alters ion channel activity, neurotransmitter release, and calcium homeostasis, contributing to excitotoxic signaling and neuronal death. PKC-driven oxidative imbalance and pro-inflammatory signaling also compromise neuron–glia communication [29,30].
- Hexosamine pathway: When fructose-6-phosphate is accumulated, it increases its flux into the hexosamine pathway, which produces an excess of UDP-N-acetylglucosamine. This overproduction leads to abnormal glycosylation of neuronal proteins and transcription factors, consequently disrupting normal gene expression and synaptic protein function. The hyperactivation of the hexosamine pathway has been associated with an increase in ROS levels, mitochondrial impairment, and reduced neuronal survival under stress conditions [29,31].
- Polyol pathway: Hexokinase enzyme saturates due to the hyperglycemic state, and aldose reductase converts the excess of glucose into sorbitol by oxidizing nicotinamide adenine dinucleotide (NAD^+^/NADH) phosphate (NADPH) to NADP^+^ [32]. Sorbitol is then metabolized by sorbitol dehydrogenase to fructose using NAD^+^ as cofactor. Sorbitol accumulation causes osmotic stress [33], while NADPH reduction impairs glutathione regeneration and increases oxidative stress. Excessive sorbitol oxidation raises the NADH/NAD^+^ ratio, which inhibits glyceraldehyde-3-phosphate dehydrogenase (GAPDH) and promotes DAG-mediated PKC activation and AGE formation [34]. Excess NADH can also fuel NADH oxidase, increasing ROS generation [28], while fructose metabolism produces potent glycation agents that further contribute to AGE [34]. Neurons express aldose reductase (particularly retinal ganglion cells), making them highly susceptible to polyol pathway activation [33].
- AGEs pathway: Chronic hyperglycemia promotes the non-enzymatic glycation of neuronal proteins and lipids. AGEs interact with RAGE, which is expressed in ganglion cells and glia, and activates oxidative and inflammatory cascades that impair neuronal viability. In Müller cells, AGEs-RAGE signaling further compromises neuronal support by promoting VEGF release, inflammation, and gliosis [glial fibrillary acidic protein (GFAP) upregulation], aggravating neurodegeneration [28,35,36,37].

Although PKC activation, polyol flux, hexosamine pathway, and AGE accumulation are all implicated in DR, their individual quantitative contribution cannot be precisely determined, as these pathways act in parallel and converge on common downstream mechanisms. Through early metabolic stress, this convergence leads to oxidative stress, mitochondrial dysfunction, osmotic imbalance, abnormal protein modifications, and inflammatory signaling. Together, these processes disrupt neuronal homeostasis, compromise synaptic function and neuronal survival at very early stages of the disease, placing neurodegeneration at the forefront of early DR, even before classical vascular abnormalities are established.

### 2.2. Oxidative Stress and Mitochondrial Dysfunction

Oxidative stress and mitochondrial dysfunction represent early and sustained events in DR. Retinal ganglion cells are particularly vulnerable due to their high metabolic demand, while Müller glia and microglia are responsible for amplifying oxidative and inflammatory responses, contributing to early neuronal dysfunction and progression of retinal neurodegeneration [38].

The activation of all the aforementioned pathways leads to ROS overproduction and redox imbalance. Meanwhile, higher glucose flux raises electron transport chain and electron leak increasing the levels of mitochondrial superoxide anion [39]. Consequently, mitochondrial dynamics (fusion/fission, mitophagy) are impaired, and damaged mitochondria are accumulated, further increasing ROS production and damaging mitochondrial DNA/lipids/proteins [40].

Independently of mitochondrial ROS production, NADPH oxidases (NOXs), especially NOX2, are upregulated and hyperactivated in retinal cells due to high glucose levels and inflammation, aggravating the redox imbalance by producing cytosolic superoxide anion [41]. Under hyperglycemic conditions, Ras-related C3 botulinum toxin substrate 1 (RAC1), a small signaling protein, becomes activated and facilitates the assembly of the NOX2 complex by recruiting its cytosolic subunits to the membrane-bound components. Once assembled, NOX2 transfers electrons from NADPH to molecular oxygen, producing superoxide anion, which can give rise to other ROS [42]. Cyclooxygenases (COXs), lipoxygenases, and xanthine oxidases also contribute to the production of cytosolic ROS [43,44,45].

Although the retinal NVU has robust antioxidant defenses, chronic hyperglycemia compromises its efficiency increasing the oxidative imbalance. These include both enzymatic (superoxide dismutase, catalase, glutathione peroxidase, etc.) and non-enzymatic antioxidants, along with repair systems that target oxidized molecules [29,46]. The reduced antioxidant activity in DR has been associated with impaired signaling of nuclear factor erythroid 2-related factor 2 (NRF2), a transcription factor that normally activates antioxidant gene expression via antioxidant response elements (DNA sequences in the promoter regions of antioxidant genes). Additionally, there are studies linking hyperglycemia with the inhibition of NRF2 expression, particularly in Müller cells, thereby promoting oxidative stress [47,48].

In parallel with mitochondrial dysfunction, increasing evidence supports a role for endoplasmic reticulum (ER) stress in diabetes-induced retinal neurodegeneration. Chronic hyperglycemia disrupts ER homeostasis, leading to persistent activation of the unfolded protein response, which amplifies oxidative stress, calcium imbalance, and pro-apoptotic signaling in retinal neurons and glial cells. Recent global analyses of research trends in retinal diseases identify ER stress as a key mechanism linking metabolic stress to retinal neurodegeneration, reinforcing its relevance in diabetic retinopathy [49].

### 2.3. Glutamate Excitotoxicity and Neurotransmitter Imbalance

Glutamate is the main neurotransmitter in the retinal visual pathway and has an essential role in synaptic transmission but becomes neurotoxic at elevated levels (excitotoxicity) by overstimulation of its receptors, leading to neuronal death. Glutamate clearance relies on Müller cell uptake via glutamate aspartate transporter 1 (GLAST) and conversion to glutamine by glutamine synthetase, maintaining the glutamate–glutamine cycle [50]. In experimental DR, it has been observed that the expression of genes involved in glutamate transport, including GLAST in Müller cells, is reduced [51,52]. Retinal ganglion cells are particularly susceptible to glutamate excitotoxicity [53].

Diabetes can also disrupt synaptic machinery, lowering the levels of presynaptic proteins such as synaptophysin, synapsin I, or syntaxin 1A, which are critical for neurotransmitter release and synaptic maintenance. These reductions have been reported in animal models and postmortem human retinas and can be attributed to a deterioration of retrograde axonal transport in retinal ganglion cells [54,55,56,57].

Together, alterations in glutamate clearance, the excitotoxicity resulting from its accumulation, and the resulting synaptic dysfunction create an environment of increased neuronal stress in the diabetic retina. These alterations are increasingly recognized as early contributing factors to the pathogenesis of RD and may occur in parallel with, or even before, detectable vascular abnormalities.

### 2.4. Inflammation and Microglial Activation

Chronic hyperglycemia triggers the initial pathological mechanisms that lead to oxidative and metabolic stress in the retina, which in turn activate inflammatory pathways that promote the release of cytokines, chemokines, and other mediators that initiate a cascade of glial cell activation. Microglia, the resident macrophages of the retina, become activated and release pro-inflammatory cytokines such as tumor necrosis factor alpha (TNF-α) and interleukin-1 β (IL-1β), contributing to retinal inflammation and neuronal damage [58,59].

Müller cells, the principal glial cells in the retina, also undergo reactive gliosis in response to hyperglycemic stress. This gliosis involves changes in gene expression and cellular morphology, which consequently alter their supportive functions, mainly glutamate uptake and potassium buffering, and promotes the release of inflammatory mediators [60]. Astrocytes, another type of glial cell, contribute to the inflammatory environment by releasing cytokines and ROS, further exacerbating neuronal injury and disrupting the BRB [61,62].

Collectively, the activation of microglia, Müller cells, and astrocytes creates a pro-inflammatory milieu that accelerates neuronal dysfunction and contributes to the early stages of neurodegeneration in DR.

Beyond associative observations, experimental studies provide mechanistic and causal evidence for the involvement of inflammation and microglial activation in neurodegeneration. Genetic modulation of key inflammatory and innate immune pathways reduces microglial activation, oxidative stress, and retinal neuronal loss in diabetic models [63]. In parallel, pharmacologic modulation of inflammatory signaling further supports a causal role for inflammation by attenuating neuronal dysfunction and neurovascular unit impairment in experimental diabetes [64]. Although direct genetic evidence in humans remains limited, these convergent findings support inflammation as an active contributor to early neuronal dysfunction in diabetic retinal disease.

### 2.5. Neurotrophic Factor Deficiency

The low retinal production of neuroprotective factors associated with diabetes compromises the capacity of arresting the neurodegenerative process. Among the factors involved are pigment epithelium-derived factor (PEDF), somatostatin (SST), interstitial retinol-binding protein (IRBP), or glucagon-like peptide-1 (GLP-1) [62,65,66,67]. Under physiological conditions, PEDF is synthesized by the retinal pigment epithelium (RPE) and plays a crucial role in retinal homeostasis by preventing oxidative stress and glutamate excitotoxicity [68]. SST, which is also mainly produced by the RPE, has antiangiogenic and neuroprotective properties [69]. Furthermore, IRBP is synthesized by photoreceptors and is essential for their survival and maintenance [70,71]. Additionally, neurotrophic factors like VEGF and erythropoietin (EPO) are overexpressed in the diabetic retina, potentially counteracting this reduction in neuroprotective factors. Other factors, including insulin, brain-derived neurotrophic factor (BDNF), glial cell line-derived neurotrophic factor (GDNF), ciliary neurotrophic factor (CNTF), and nerve growth factor (NGF) might also have an important role in the neurodegenerative process that occurs in DR [62,72,73]. In fact, BDNF has emerged as a key regulator of retinal neuronal survival and synaptic plasticity. A recent systematic review integrating clinical, pathological, and experimental evidence demonstrated that BDNF levels are consistently altered in diabetic retinopathy, particularly at early stages, and are associated with retinal neurodegeneration and functional impairment rather than with advanced vascular pathology [74]. These findings support the concept that impaired BDNF signaling contributes to early neuronal dysfunction in diabetic retinal disease and highlight its potential value as both a biomarker and a therapeutic target.

### 2.6. Vascular–Neuronal Interactions (Neurovascular Unit Dysfunction)

NVU integrates neurons, endothelial cells, and pericytes to ensure homeostasis, coupling neuronal activity with vascular supply, and preserving BRB integrity [7]. In DR, hyperglycemia and oxidative stress can directly affect vascular cells too, causing early endothelial dysfunction and pericyte dropout, even before that microvascular lesions can clinically be detected [75,76]. Pericyte loss destabilizes retinal capillaries, impairs endothelial survival signaling, and disrupts proper myogenic response, making neurons more vulnerable to changes in blood flow and pressure [77].

Endothelial injury is characterized by downregulation of tight-junction proteins such as occludin and claudin-5, which increases BRB permeability and facilitates leakage of plasma proteins into the retinal parenchyma [78]. These extravasated molecules exert direct neurotoxic effects, amplifying oxidative stress and promoting neuronal apoptosis [79]. At the same time, neuronal hyperactivity and metabolic stress increase oxygen demand, but impaired functional hyperemia interferes with the ability of retinal blood vessels to adapt adequately to blood flow, leading to hypoxia and accelerating neuronal dysfunction [15].

This interplay establishes a vicious cycle: vascular instability compromises neuronal survival, while stressed or dying neurons release vasoactive and pro-apoptotic mediators, such as glutamate and nitric oxide, which further damage endothelial cells and pericytes [7,80]. Glutamate accumulation has been shown to impair vascular integrity, while vascular leakage introduces neurotoxic agents like fibrinogen into the neural retina, worsening neurodegeneration [50,80,81]. Therefore, vascular impairment within the NVU not only occurs in parallel to neuronal dysfunction, but it also actively exacerbates the process, consequently creating a self-reinforcing loop that accelerates the progression of the disease.

**Figure 1:**
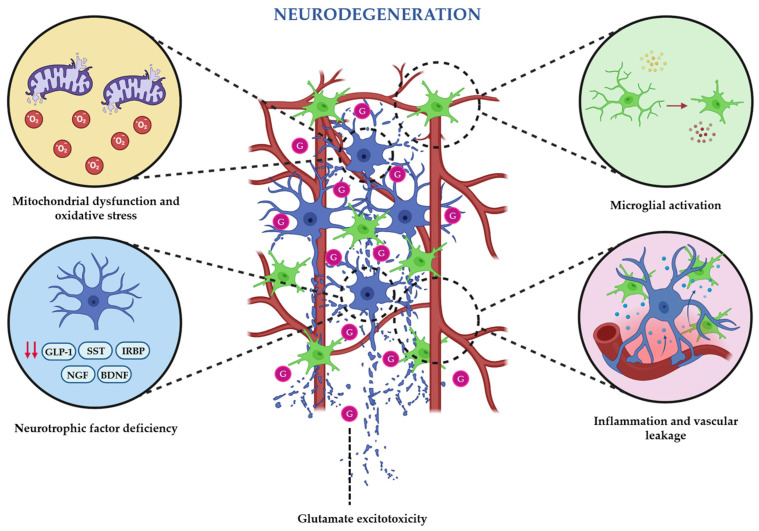
Schematic illustration of the main pathophysiological mechanisms that trigger and maintain the neurodegenerative state that occur during the earliest stages of diabetic retinopathy.

In summary, all the addressed pathophysiological mechanisms illustrate that chronic hyperglycemia initiates a cascade of metabolic, oxidative, and inflammatory alterations that trigger early retinal neurodegeneration, while excitotoxicity and neurotrophic support deficiencies further compromise neuronal integrity (Figure 1). As vascular instability and BRB breakdown emerge, neuronal and vascular dysfunctions reinforce each other, ultimately resulting in a progressive damaging neurovascular cycle that ultimately accelerates the development of DR.

## 3. Retinal Phenotyping in Diabetic Patients

DR has been classified by the vascular abnormalities, which are visible by means of direct or indirect fundus exam [81]. Currently two classifications are used: the Early Treatment of Diabetic Retinopathy Study (ETDRS) classification [82], which is used in clinical trials and research and the International Clinical Diabetic Retinopathy (ICDR) Severity Scale, which is a simplification of the first one and more usable in clinical practice. Advances in imaging technology and in the understanding of DR pathogenesis urges for new phenotyping approaches [83].

New retinal imaging approaches like Optical Coherence Tomography (OCT) have contributed to characterizing the diabetic macular edema (DME). OCT biomarkers have been proposed to predict the response to different treatments in diabetic macular edema [84,85]. For example, the disorganization of the retinal inner layers [86] or the disruption of the external limiting membrane [87] have been associated with poorer visual acuity responses to treatment. Furthermore, cytokines and inflammatory markers in serum, vitreous or even aqueous humor have been proposed to determine the prognosis and the responses to treatment of DME [88]. In addition, the assessment of different biomarkers in the aqueous humor has been proposed to predict the response to the two main therapeutic options in DME: corticosteroids (for those subjects with more inflammatory markers) or anti-VEGF (when the expression of VEGF dominates) [89]. However, more prospective studies are needed to implement all this information in the clinical practice towards a personalized medicine.

Regarding the pathogenesis of DR, it is now not only considered a merely vascular disease, but a neurovascular disease where neurodegeneration plays an important role. Neurodegeneration can be detected before vascular changes are evident by OCT or by functional tests like electroretinogram. In fact, new approaches for early treatment of DR include the use of neuroprotective agents [65]. Simó et al. conducted a clinical trial designed to arrest the progression of DR in early stages using eye drops of somatostatin (the European Consortium for Early Treatment of Diabetic Retinopathy or EUROCONDOR trial). One of the main lessons of this trial was that neurodegeneration (at least when assessed by multifocal electroretinography) was not identified in a significant proportion (35%) of patients with early microvascular impairment (ETDRS 20-34) [90,91]. Thus, neurodegeneration may represent an early indicator of diabetic retinopathy only in a specific group of individuals with diabetes. This observation underscores the importance of screening for retinal neurodysfunction to identify patients who could benefit from neuroprotective therapies. The future implications of these two phenotypes have not yet been explored. In fact, there is an initiative towards a proposal of a new approach for the staging of DR that considers the presence of neurodegeneration and macular edema [92].

In the future, the impact of genome studies or the analysis of OCT and/or retinal images by AI could contribute to the definition of new retinal phenotypes that could not only been related to DR but also to systemic diseases such as dementia or cardiovascular disease [93,94].

## 4. Therapeutic Strategies Based on Neuroprotection

### 4.1. New Emerging Neuroprotective Agents

Neurodegeneration is now recognized as an early and key component of DR, often preceding microvascular lesions and contributing to inflammation, vascular leakage, and vision loss [5,95]. Multiple experimental studies have evidenced the efficacy of promising neuroprotective strategies targeting different stages of the neurodegenerative process, such as oxidative stress, synaptic impairment, or neurotrophic factors deficiency, among others [5,95]. However, translation to patients has been limited [15,91,96,97]. A key reason is that clinical trials and regulatory endpoints have focused almost exclusively on vascular changes, ignoring neurodegeneration. Updating the DR staging system to include neuronal damage could pave the way for trials that adequately evaluate neuroprotective therapies and allow them to complement existing treatments focused on the vascular components of the disease [98,99,100].

The search for neuroprotective therapies in DR has grown considerably in the past decade, moving beyond glucose control and vascular endpoints to focus on mechanisms that directly benefit retinal neurons [15,65]. A mechanistic classification provides a clearer framework for understanding how diverse agents intervene in the complex pathophysiology of early DR and converge to drive neurodegeneration.

#### 4.1.1. Antioxidants and Mitochondrial Protectors

ROS and mitochondrial dysfunction have a key role in neuronal injury in DR. Classical antioxidants such as α-lipoic acid (ALA) improve mitochondrial function, reduce oxidative damage, and preserve retinal structure and electrophysiology in experimental diabetes [101,102]. Mitochondria-targeted antioxidants such as SkQ1 selectively accumulate within mitochondria and prevent ganglion cell loss, preserving retinal integrity in diabetic and senescence-accelerated rat models [103]. Pharmacological activation of NRF2, the main regulator of antioxidant defenses, has exhibited promising results too. Sulforaphane restored antioxidant enzyme activity and improved retinal function in diabetic models through NRF2-dependent mechanisms [104]. Together, these strategies aim not only to scavenge ROS but to restore mitochondrial redox balance and prevent neuronal apoptosis.

#### 4.1.2. Therapeutic Approaches Targeting the Reduction in Excitotoxicity and the Preservation of Synapses

The excitotoxicity caused by glutamate accumulation is one of the main contributors of retinal neuronal loss in diabetes. Brimonidine, an α2-adrenergic agonist, can inhibit this glutamate accumulation and its detrimental effects by reducing glutamate release and activating pro-survival mechanisms (PI3K/Akt, ERK). In the EUROCONDOR trial, topical brimonidine preserved retinal function in patients with early DR [91,105]. Citicoline (CDP-choline) is able to stabilize neuronal membranes, enhance mitochondrial bioenergetics, and improve pattern electroretinogram responses, delaying neurodegeneration in small human and animal studies [106,107,108]. NMDA receptor antagonists, such as memantine, attenuate calcium influx and excitotoxic damage, demonstrating neuroprotection in diabetic rodents, although systemic side effects have limited clinical translation [109]. All these treatments aim to maintain synaptic integrity and prevent early neuronal dysfunction.

#### 4.1.3. Anti-Inflammatory and Immunomodulatory Agents

Inflammation amplifies the neurodegenerative cascades of DR. SOCS1-derived peptides, when applied topically, inhibit JAK/STAT signaling, and are able to prevent glial activation and vascular leakage in diabetic models [110]. Minocycline is an antibiotic modulator of the microglia that decreases TNF-α, IL-1β and oxidative stress, resulting in the protection of retinal neurons in diabetic rodents [111]. Palmitoylethanolamide acts on peroxisome proliferator-activated receptor alpha (PPAR-α) to suppress glial reactivity and inflammation, improving retinal structure and function in experimental diabetes [112]. In addition, the inhibition of P2X7 purinergic receptors, which are mediators of inflammasome activation, reduces neuronal apoptosis in the diabetic retina [113]. These results support that inflammation control is a relevant neuroprotective approach in DR.

#### 4.1.4. Vasoactive and Neurovascular Protectors

Given the close interrelationship of neuronal and vascular compartments, therapies that improve microvascular health can also confer neuroprotection. Calcium dobesilate is a vasoactive and antioxidant compound that has demonstrated efficacy in reducing vascular permeability, oxidative stress, and excitotoxicity in the diabetic retina, although clinical results are heterogeneous [114]. Another neurovascular protector is bosentan, an endothelin receptor antagonist, which improves retinal perfusion and reduces neuronal cell death in diabetic models, indicating that retinal blood flow normalization can indirectly protect neurons [115].

#### 4.1.5. Therapies Based on Neurotrophic and Growth Factors

Loss of neurotrophic support is a hallmark of DR. Somatostatin, normally secreted by retinal neurons, is markedly reduced in diabetes; topical somatostatin analogs have shown neuroprotective effects both experimentally and in the EUROCONDOR clinical trial [91,105].

CNTF and BDNF promote retinal ganglion cell survival in diabetic models when delivered via encapsulated-cell technology or viral vectors [116]. PEDF, an anti-angiogenic and neuroprotective protein downregulated in DR, prevents oxidative stress and apoptosis when supplemented topically [117]. NGF eye drops prevent neurodegeneration in experimental diabetes [118]. Erythropoietin (EPO), beyond its hematopoietic role, protects against neuronal apoptosis when delivered locally to the retina [119].

GLP-1 and its receptor (GLP-1R) form an endogenous neurotrophic system in the retina. GLP-1 and GLP-1R expression is downregulated in diabetic retina, contributing to increased oxidative stress and neuronal vulnerability [66]. Activation of GLP-1R with liraglutide or exendin-4 triggers PI3K/Akt and CREB survival pathways upregulates BDNF and reduces apoptosis and gliosis [66,120,121].

Enhancing endogenous GLP-1 signaling through DPP-4 inhibitors, such as sitagliptin, has also demonstrated retinal neuroprotection [117,118,119]. Recent studies showed that sitagliptin eye drops reach the retina and prevent neurovascular unit impairment without systemic effects (glycemia and body weight) [122,123,124]. In addition, GLP-1R activation has been found to prevent the downregulation of presynaptic proteins involved in vesicle biogenesis and mobilization in diabetic retinas. Additionally, GLP-1R has shown to protect against diabetes-induced retinal thinning. These neuroprotective effects are associated with the improvement of retinal function assessed by electroretinography (ERG). To date, retinal neuroprotection by GLP-1R agonists and DPP-4 inhibitors has been demonstrated only in experimental models. Systemic therapies provide indirect translational support, whereas topical formulations remain preclinical [122,123,124].

Beyond retinal experimental models, recent narrative reviews highlight that GLP-1 receptor agonists exert pleiotropic neuroprotective, anti-inflammatory, and antioxidant effects across multiple diabetes-associated comorbidities, reinforcing the translational relevance of GLP-1R activation as a clinically attractive pathway for retinal neurodegeneration [125].

These findings support GLP-1 as a key neurotrophic and metabolic factor whose replacement may prevent early neuronal loss in DR.

Table 1 summarizes neuroprotective candidates investigated in early DR, highlighting their preclinical neuroprotective effects, primary mechanisms of action, cellular targets, and stage of evaluation.

### 4.2. Novel Delivery Routes

Effective retinal delivery of neuroprotective agents remains a major challenge due to ocular barriers, including the cornea, sclera, and BRB. To overcome these limitations, several novel drug delivery systems have been investigated to enhance retinal bioavailability, prolong therapeutic effects, and reduce systemic exposure.

Intravitreal injections are the most direct route to deliver drugs to the retina, allowing high local concentrations of neuroprotective agents. However, the invasiveness of this delivery route has been associated with adverse effects including endophthalmitis, retinal detachment, and patient discomfort. To overcome these issues, sustained-release intravitreal implants have been developed, which can continuously release therapeutic agents over weeks to months, reducing the frequency of injections while maintaining effective drug levels. For instance, biodegradable implants have been studied for the delivery of corticosteroids and neurotrophic factors, demonstrating prolonged retinal exposure and neuroprotective effects [126].

Topical eye drops, traditionally limited by poor penetration to the posterior segment, have gained renewed interest through novel formulations [127]. One of the innovative strengths of this route is that it allows the drug to be delivered in a much safer way without losing efficacy. In addition, it allows self-administration, reducing visits, and medical interventions [128]. However, errors in self-administration of eye drops may occur, increasing the risk of therapeutic failure. Despite this, with proper patient education on long-term administration of eye drops this issue can be overcome [129].

Another innovative approach that has recently emerged is the suprachoroidal delivery, which consists of injecting drugs into the space between the sclera and choroid. This technique achieves high posterior segment drug concentrations while minimizing exposure to the anterior segment and systemic circulation. It is particularly suitable for delivering biologics and large-molecule neuroprotective agents, providing targeted and sustained retinal exposure [130].

Carriers based on nanotechnology have also been widely explored for retinal neuroprotection. Liposomes, dendrimers, and polymeric nanoparticles can encapsulate both hydrophilic and hydrophobic drugs and protect them from degradation and enabling controlled release. The engineering of magnetic nanoparticles has allowed targeted delivery with external guidance to all retinal layers for localized treatment. These strategies enhance the stability, bioavailability, and efficacy of neuroprotective agents [130].

Thermoresponsive hydrogels are an emerging drug delivery system designed for the posterior segment of the eye. These materials are liquids at room temperature, allowing injection through fine needles and quickly form gels at body temperature, creating a localized drug reservoir. This gel gradually releases therapeutic agents, such as anti-VEGF proteins, enabling sustained treatment over weeks or months. This approach addresses the limitations of frequent intravitreal injections, reducing complications and improving patient compliance. By controlling polymer composition and gel properties, drug release can be finely tuned, making thermoresponsive hydrogels a promising minimally invasive platform for chronic retinal disease management [131].

Stem cell-based therapies offer a biological method of drug delivery, as transplanted stem cells can secrete neurotrophic factors locally within the retina. Intravitreal or subretinal transplantation allows sustained release of neuroprotective agents, potentially repairing or preserving retinal neurons in diabetic eyes [132].

Finally, biodegradable microparticles have been investigated as vehicles for sustained drug delivery. These particles encapsulate neuroprotective agents and release them gradually as the polymer degrades, providing controlled and prolonged retinal exposure when administered intravitreally [127].

In summary, the development of novel ocular delivery routes, ranging from intravitreal implants and eye drops to suprachoroidal injections, nanocarriers, thermoresponsive hydrogels, and stem cell-based systems, offers promising strategies for neuroprotection in DR. By overcoming ocular barriers, enabling targeted and sustained release, and minimizing systemic side effects, these approaches have the potential to preserve retinal function, reduce treatment burden, and improve long-term visual outcomes.

### 4.3. Personalized Medicine: Patient Stratification and Targeted Therapies

Personalized neuroprotection in DR addresses the heterogeneity of retinal neurodegeneration, which often precedes visible vascular damage, by adapting interventions based on functional assessments, advanced imaging, and systemic or ocular biomarkers [133,134]. However, several limitations make widespread implementation difficult. Reliable and standardized biomarkers for early neurodegeneration are still lacking, and many functional or imaging techniques, such as ERG and OCT angiography, may not be routinely available in all clinical settings [135,136]. Stratifying patients accurately requires longitudinal data and complex interpretation, which can be resource-intensive. Furthermore, evidence from animal models demonstrates early neuronal dysfunction and synaptic alteration before cell death, but translation to human disease remains uncertain [137,138]. The long-term safety, optimal dosing, and sustained efficacy of many neuroprotective agents, particularly the newest therapies, are not yet established [91]. Patient adherence, comorbidities, and variability in metabolic control further complicate individualized strategies. Finally, integrating personalized neuroprotection into routine practice demands robust predictive models and clinical decision support systems, which are not yet fully developed [139]. Despite these challenges, targeted approaches combining neuroprotection with metabolic management and lifestyle interventions hold promise for preserving retinal neuronal function and preventing vision loss in high-risk patients. 

## 5. Future Perspectives and Unmet Needs

Future advances in neuroprotection for DR will rely on the capacity of integrating retinal phenotyping with molecular biomarkers, allowing early detection and precise patient stratification. By combining advanced imaging tools, such as OCT angiography and adaptive optics, with biomarkers such as neurofilament light chain, GFAP, or AGEs, it may be possible to distinguish between patients with predominantly neurodegenerative or vascular phenotypes, thereby facilitating individualized treatment [134,135]. Another critical need is the redesign of clinical trials to focus on neuronal endpoints. Most current studies emphasize vascular outcomes, whereas early neuronal dysfunction often predicts visual decline. Future trials should incorporate multimodal endpoints that combine functional (ERG, microperimetry) and structural (OCT) measures, alongside molecular indicators of neuroinflammation and oxidative stress [91,140]. Additionally, a shift toward combined vascular and neuronal therapeutic approaches is essential, recognizing that the NVU functions as an integrated system. Dual-target strategies, pairing anti-VEGF, or vascular stabilizers with neurotrophic or anti-inflammatory agents would provide synergistic benefits and prevent irreversible vision loss [5,137]. Overall, advancing toward a precision medicine model that unites imaging, molecular diagnostics, and computational prediction represents the most promising path to address the unmet needs of neuroprotection in DR.

## Figures and Tables

**Table 1:** Summary of agents that have demonstrated neuroprotective effects in experimental models of early DR. Agents are listed in the order of appearance in Section 4.

Neuroprotective Agent	Primary Mechanism of Action	Evidence/Trial Status
α-Lipoic acid (ALA)	Antioxidant activity and mitochondrial function preservation	Preclinical
SkQ1	Mitochondria-targeted antioxidant activity	Preclinical
Sulforaphane	Activation of endogenous antioxidant defenses (NRF2 pathway)	Preclinical
Brimonidine	Reduction in glutamate excitotoxicity and activation of neuronal survival signaling	Phase II/III clinical trial; no significant effect on primary endpoint
Citicoline (CDP-choline)	Membrane stabilization and support of synaptic and mitochondrial function	Preclinical; small exploratory human studies
Memantine	NMDA receptor antagonism; reduction in excitotoxicity	Preclinical
SOCS1-derived peptides	Inhibition of cytokine signaling and glial activation	Preclinical
Minocycline	Modulation of microglial activation and inflammatory signaling	Preclinical
Palmitoylethanolamide	PPAR-α-mediated anti-inflammatory signaling	Preclinical
P2X7 receptor inhibitors	Suppression of inflammasome-related signaling	Preclinical
Calcium dobesilate	Improvement of microvascular function and reduction in oxidative stress	Controlled trials; evidence on vascular outcomes, not designed for neuroprotection
Bosentan	Endothelin receptor antagonism and improvement of retinal perfusion	Preclinical
Somatostatin analogs	Restoration of neurotrophic signaling and modulation of neuronal activity	Phase II/III clinical trial; no significant effect on primary endpoint
CNTF	Neurotrophic support promoting retinal ganglion cell survival	Preclinical/early-phase clinical studies
BDNF	Neurotrophic support and synaptic maintenance	Preclinical
PEDF	Anti-angiogenic and neuroprotective activity	Preclinical
NGF	Neurotrophic support and neuronal survival	Preclinical
Erythropoietin (EPO)	Anti-apoptotic and neuroprotective signaling	Preclinical
GLP-1 receptor agonists (topical)	Metabolic and neurotrophic signaling enhancing neuronal survival	Preclinical
DPP-4 inhibitors (topical)	Enhancement of endogenous GLP-1 signaling and neuroprotection	Preclinical

## Data Availability

No new data were created or analyzed in this study. Data sharing is not applicable to this article.

## Abbreviations

The following abbreviations are used in this manuscript:

AGEs	Advanced Glycation End-products
ALA	α-Lipoic Acid
ATP	Adenosine Triphosphate
BDNF	Brain-Derived Neurotrophic Factor
BRB	Blood–Retinal Barrier
CDP-choline	Cytidine Diphosphate Choline
CNTF	Ciliary Neurotrophic Factor
COX	Cyclooxygenase
CREB	cAMP Response Element-Binding Protein
DAG	Diacylglycerol
DME	Diabetic Macular Edema
DPP-4	Dipeptidyl Peptidase-4
DR	Diabetic Retinopathy
EPO	Erythropoietin
ER	Endoplasmic Reticulum
ERG	Electroretinography
ETDRS	Early Treatment of Diabetic Retinopathy Study
EUROCONDOR	European Consortium for Early Treatment of Diabetic Retinopathy
GAPDH	Glyceraldehyde-3-Phosphate Dehydrogenase
GDNF	Glial Cell Line-Derived Neurotrophic Factor
GFAP	Glial Fibrillary Acidic Protein
GLAST	Glutamate Aspartate Transporter 1
GLP-1	Glucagon-Like Peptide-1
GLP-1R	Glucagon-Like Peptide-1 Receptor
ICDR	International Clinical Diabetic Retinopathy
IL-1β	Interleukin-1 Beta
IRBP	Interstitial Retinol-Binding Protein
JAK/STAT	Janus Kinase/Signal Transducer and Activator of Transcription
NMDA	N-Methyl-D-Aspartate
NOX	NADPH Oxidase
NOX2	NADPH Oxidase 2
NRF2	Nuclear Factor Erythroid 2–Related Factor 2
NVU	Neurovascular Unit
OCT	Optical Coherence Tomography
PEDF	Pigment Epithelium-Derived Factor
PI3K/Akt	Phosphoinositide 3-Kinase/Protein Kinase B
PKC	Protein Kinase C
PPAR-α	Peroxisome Proliferator-Activated Receptor Alpha
RAC1	Ras-Related C3 Botulinum Toxin Substrate 1
RAGE	Receptor for Advanced Glycation End-products
RD	Retinal Disease
RGCs	Retinal Ganglion Cells
RPE	Retinal Pigment Epithelium
ROS	Reactive Oxygen Species
SkQ1	Mitochondria-Targeted Antioxidant (Plastoquinone Derivative)
SOCS1	Suppressor of Cytokine Signaling 1
SST	Somatostatin
TNF-α	Tumor Necrosis Factor Alpha
UDP	Uridine Diphosphate
VEGF	Vascular Endothelial Growth Factor
